# Transcriptomic response of bioengineered human cartilage to parabolic flight microgravity is sex-dependent

**DOI:** 10.1038/s41526-023-00255-6

**Published:** 2023-01-19

**Authors:** A. K. Aissiou, S. Jha, K. Dhunnoo, Z. Ma, D. X. Li, R. Ravin, M. Kunze, K. Wong, A. B. Adesida

**Affiliations:** 1 Department of Medicine, Faculty of Medicine & Dentistry, University of Alberta, Edmonton, AB Canada; 2 Department of Mechanical Engineering, Faculty of Engineering, University of Alberta, Edmonton, AB Canada; 3 Department of Surgery, Faculty of Medicine & Dentistry, University of Alberta, Edmonton, AB Canada; 4 Department of Civil & Environmental Engineering, University of Alberta, Edmonton, AB Canada; 5 Institute for Aerospace Studies, Faculty of Applied Science & Engineering, University of Toronto, Toronto, ON Canada; 6 Department of Chemical and Materials Engineering, Faculty of Engineering, University of Alberta, Edmonton, AB Canada

**Keywords:** Stem cells, Computational biology and bioinformatics

## Abstract

Spaceflight and simulated spaceflight microgravity induced osteoarthritic-like alterations at the transcriptomic and proteomic levels in the articular and meniscal cartilages of rodents. But little is known about the effect of spaceflight or simulated spaceflight microgravity on the transcriptome of tissue-engineered cartilage developed from human cells. In this study, we investigate the effect of simulated spaceflight microgravity facilitated by parabolic flights on tissue-engineered cartilage developed from in vitro chondrogenesis of human bone marrow mesenchymal stem cells obtained from age-matched female and male donors. The successful induction of cartilage-like tissue was confirmed by the expression of well-demonstrated chondrogenic markers. Our bulk transcriptome data via RNA sequencing demonstrated that parabolic flight altered mostly fundamental biological processes, and the modulation of the transcriptome profile showed sex-dependent differences. The secretome profile analysis revealed that two genes (*WNT7B* and *WNT9A*) from the Wnt-signaling pathway, which is implicated in osteoarthritis development, were only up-regulated for female donors. The results of this study showed that the engineered cartilage tissues responded to microgravity in a sex-dependent manner, and the reported data offers a strong foundation to further explore the underlying mechanisms.

## Introduction

Mechanical stimulation is crucial for cartilage development, and the health of cartilage tissue is maintained by the intricate balance between loading and unloading forces^1^. Chondrocytes, the cells responsible for making cartilage, can sense environmental perturbations through their surrounding pericellular matrix (PCM), which is composed primarily of perlecan, aggrecan, hyaluronan, and minor collagens^2,3^. These mechanical stimuli are transmitted through the PCM to the encapsulated chondrocytes, where they are detected by receptors, molecular Ca^2+^ channels, primary cilium, and integrins on the cell membrane. The physical signals are then converted into biological and biochemical signals by triggering a cascade of downstream activities^4^.

In weight-bearing joints such as the hip and knee, cartilage is under continuous mechanical loading. It is well established that an appropriate amount of loading can increase cartilage thickness, proteoglycan content, and improve the mechanical properties^5–8^. However, prolonged absence of mechanical loading can induce excessive catabolic activities leading to cartilage atrophy and is one of the major causes of osteoarthritis (OA)^9^. For example, mechanical unloading via knee joint immobilization of ten healthy individuals (4 males and 6 females) with no history of OA resulted in magnetic resonance imaging parameters that resembled that of knee OA^10^. Further, it has been observed that a prolonged period of off-loading results in an overall decrease in articular cartilage mass and inhibits the early stages of cartilage formation^11^. Thus, it is reasonable to suggest that chondrocytes can sense and respond to both mechanical loading and unloading signals.

To investigate the effect of mechanical unloading on anabolic and catabolic activities in cartilage, microgravity (the near absence of the forces of gravity) was proposed to create this off-loaded state^12,13^. One way to achieve microgravity is through simulation with devices in ground-based facilities such as the random positioning machine (RPM), fast-rotating clinostats, and the rotating wall vessel (RWV)^14,15^. Another, more physically realistic method for microgravity is through cell cultures performed in outer space, like at the International Space Station, or through short-term exposure to frictionless freefall from parabolic flight maneuvers^16^.

Several studies have been conducted in either real or simulated microgravity on cartilage tissues to understand its influence on a molecular and genetic basis. At the cellular level, long-term exposure to a microgravity environment has been reported to induce the increased expression of OA-related structural and signaling molecules^17,18^. At the tissue level, microgravity was shown to significantly reduce cartilage extracellular matrix density and thickness in mice^19,20^. Additionally, Freed et al. conducted one of the longest cell culture experiments in real microgravity and found that the four-month culture in the Mir space station produced mechanically inferior cartilage as compared to those cultured on Earth^21^. Interestingly, the first molecular signals to trigger these types of macrophenomena can be detected very early, after only minutes of microgravity exposure^16^. Short periods of microgravity exposure produced by parabolic flight maneuvers have been shown to alter gene expression patterns of human chondrocytes as compared to a gravity-dependent, load-bearing state^22,23^. Further, studies have shown that cartilage and meniscus tissues derived from male and female donors behave differently in a sex-dependent manner when exposed to microgravity^24,25^.

There are many intrinsic factors that may contribute to baseline sex differences in relation to OA development, particularly anatomical structure variations, gait kinematics, and hormonal differences. Wise et al. examined a cohort of patients ages 45–79 with symptomatic knee OA (KOA) and found a sex-dependent difference in the distal femur and proximal tibia shapes, with females having significantly narrower distal femoral condyle width^26^. This difference in bone anatomical structure was indicated as a potential risk factor for KOA development^26^. Furthermore, Kari et al. investigated sex-specific gait kinematics in healthy individuals in response to symmetric military loads and found a great peak hip abduction angle for females in the unloaded and medium load conditions^27^. They suggested that this difference in gait adaptation to load may contribute to the increased risk of hip or knee injuries in females^27^. Finally, several studies examined sex-dependent hormonal differences and their effect on OA development^28–32^.

To our knowledge, the sex-dependent response of chondrocytes to real microgravity has not been adequately characterized at the transcriptome level. Furthermore, sex-dependent response to short time (minutes) microgravity has not been thoroughly examined. In this study, we hypothesize that the transcriptome of tissue-engineered human cartilage will be perturbed by short-term microgravity via parabolic flight maneuvers in a sex-dependent manner.

## Methods

### Ethics statement and human bone marrow aspirate collection

Human bone marrow aspirates with non-identifying information of donors were collected from the Misericordia and University of Alberta Hospital. The methods were performed in accordance with relevant guidelines, regulations, and approval of the University of Alberta’s Health Research Ethics Board-Biomedical Panel (Study ID: Pro00018778). Non-identifying donor information is listed in Table 1.

**Table 1:** Non-identifying donor information.

Sex	Donor Number	Age	Population Doubling (PD)
Male	M1	57	16.20
	M2	64	11.52
	M3	58	16.00
Female	F1	64	13.34
	F2	59	16.45
	F3	64	15.74

### Isolation and expansion of human bone marrow mesenchymal stem cells (hBMSC)

Human bone marrow aspirates were collected from the iliac crest of three male (ages 57–64) and three female (ages 59–64) donors. The donors’ basic information is summarized in Table 1. The mononucleated cells (MNCs) of the aspirates were obtained after centrifugation on sterile-filtered Histopaque®-1077 (Millipore Sigma) according to the manufacturer’s instruction. The isolated MNC were plated in T150 cm^2^ flask at 1 × 10^5^ cells/cm^2^ in Alpha Modification – Minimum Essential Medium Eagle (α-MEM) supplemented with 10% v/v fetal bovine serum (FBS), 100 mM 4-(2-hydroxyethyl)-1-piperazineethanesulfonic acid (HEPES), 1 mM sodium pyruvate (Sigma-Aldrich Co., MO, USA), 100 U/mL penicillin, 100 lg/mL streptomycin, 0.29 mg/mL glutamine (PSG; Life Technologies, ON, Canada), and 5 ng/mL fibroblast growth factor 2 (FGF-2) (Neuromics, MN, USA, Catalog#: PR80001). The isolated MNC were then expanded in the medium described above with 5 ng/mL FGF-2) to maintain the chondrogenic potential of the cells in normoxia (NRX; 21% O_2_) at 37 °C in a humidified incubator with 5% CO_2_. The nucleated cells grew and adhered for seven days before the first medium change. After this the medium was changed twice each week until the cells were 80% confluent. The adherent hBMSC were then detached using 0.05% w/v trypsin-Ethylenediaminetetraacetic acid (trypsin-EDTA, Corning, Mediatech Inc. VA, USA) and expanded until passage 2 (P2) as previously described^33^.

### Colony-forming unit fibroblastic (CFU-F) assay

We performed a colony-forming unit fibroblastic assay to ascertain the clonogenic and population doubling characteristics of the hBMSC. We plated MNC 1 × 10^5^ MNC per 100 mm sterile Petri dishes (Becton Dickinson, ON, Canada). The plating was performed in triplicates and cultured under NRX conditions with α-MEM supplemented with 10% v/v heat-inactivated FBS, PSG, HEPES, sodium pyruvate, and 5 ng/mL FGF-2 (as above). After one week, the non-adherent population was removed by aspiration and the medium was changed twice each week. The culture time used for each hBMSC donor was the time needed to reach 80% confluence at P0, and subsequent detachment and splitting to P1 for expansion. The cell colonies were fixed with 10% w/v buffered formalin (3.8% w/v formaldehyde, Anachemia Canada Co, QC, Canada), rinsed with phosphate-buffered saline (PBS, Sigma-Aldrich), and stained with 0.25% w/v crystal violet. We then recorded the number and determined the diameter of the colonies formed. The number of colonies was used to determine the cell population doubling (CPD; Table 1) of hBMSC as described by Solchaga et al.^34^.

### In vitro chondrogenic differentiation in type I collagen porous scaffolds

**Fig. 1:**
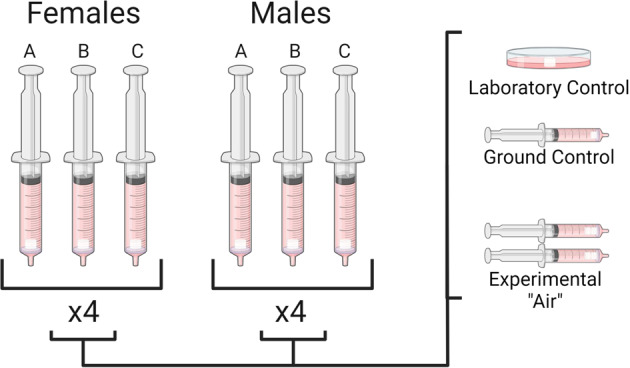
Illustration of the experimental (Air) and control groups used in this study. There is a total of six donors (three male, three female). Each donor was divided into four subgroups, including one laboratory control, one ground control, and two experimental groups.

To form tissue-engineered cartilage constructs, we seeded type I collagen porous sponge scaffolds (Integra Lifesciences Co., NJ, USA) with P2 hBMSC at a fixed density of 5 × 10^6^ cells/cm^3^ as described previously^21^. Before seeding, the dry scaffolds were 3.5 mm (height) and 6 mm (diameter). The constructs were cultured in a defined serum-free chondrogenic medium containing high glucose Dulbecco’s Modified Eagles’ Medium (high glucose DMEM, Sigma-Aldrich), 100 units/mL penicillin, 100 μg/mL streptomycin, 2 mM L-glutamine, 10 mM HEPES (Sigma-Aldrich) (all others from Life Technologies), ITS + 1 premix (Corning, Discovery Labware, Inc., MA, USA), 100 nM dexamethasone, 365 μg/mL ascorbic acid 2-phosphate, 125 μg/mL human serum albumin and 40 μg/mL L-proline (all from Sigma-Aldrich) and 10-ng/mL transforming growth factor-beta 3 (TGF-β3, Prospec, NJ, USA, Catalog#: cyt-113) as described previously in 24-well plate^35^. Each construct received 1 mL chondrogenic medium, which we replaced twice a week for a total culture period of 17 days under normoxic conditions. A total of six constructs were prepared per hBMSC donor. The six constructs were distributed into three groups: one construct/laboratory control (Lab), one construct/ ground control (Ground), and four constructs per parabolic flight (Experimental, Air). The four experimental constructs were further divided into two such that there were two experimental groups with two constructs in a single group (Fig. 1). All constructs were kept in serum-free chondrogenic media. The constructs in the laboratory control group were kept in chondrogenic media under normoxic culture conditions as before until the ground control, and parabolic flight constructs were terminated by transfer into RNAlater. The total duration that all groups were in chondrogenic media was 21 days.

### Engineered syringe apparatus

**Fig. 2:**
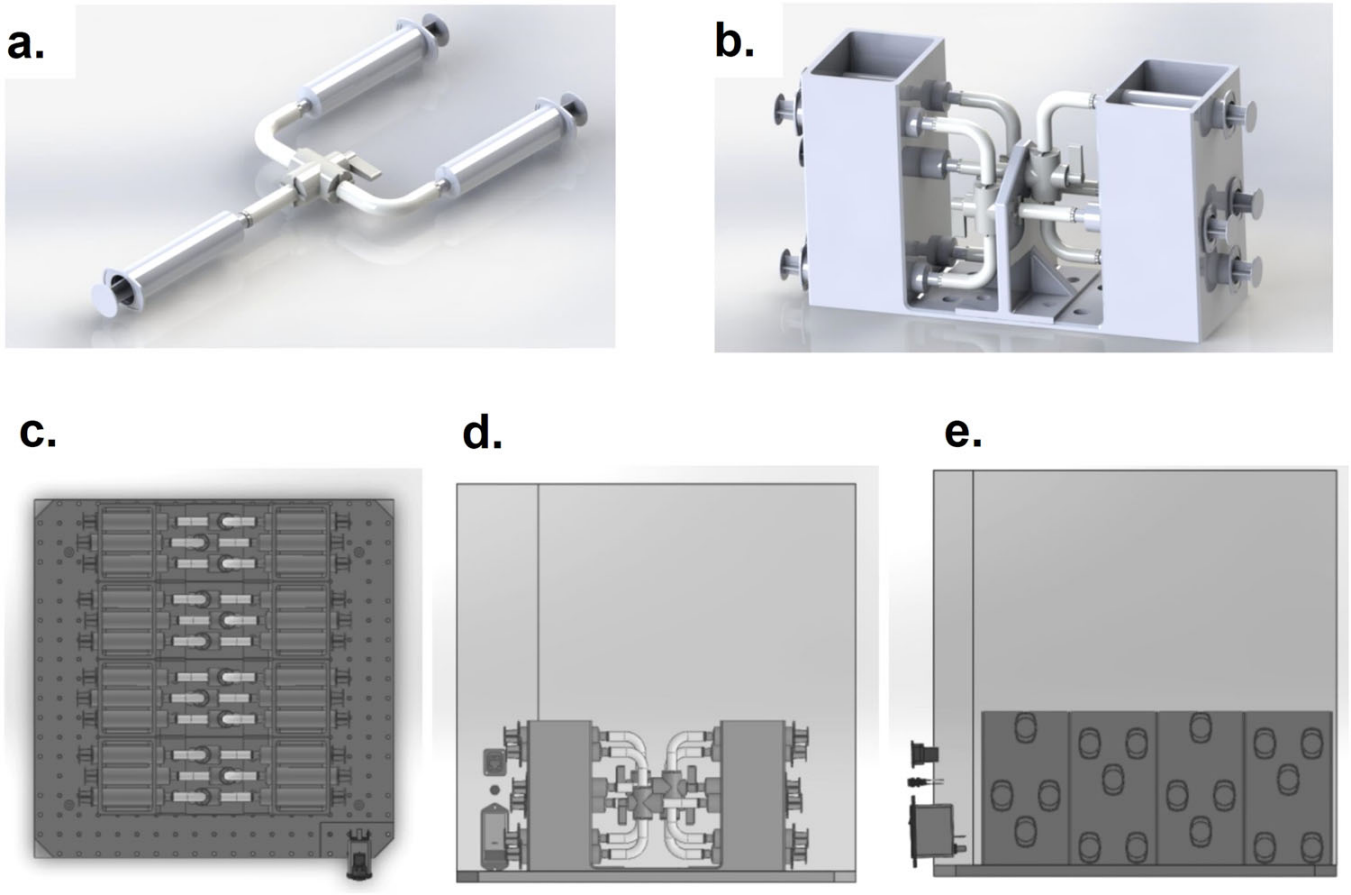
Rendering of the syringe system with or without a pelican case. **a** 3D rendering of one syringe system sub-unit containing three syringes connected by tubing and 3-position valve. **b** 3D rendering of one syringe system unit containing three syringe system sub-units secured in a 3D-printed frame. **c** 2D rendering of the syringe system in the pelican case as viewed from the top. **d** 2D rendering of the syringe system in the pelican case as viewed from the side. **e** 2D rendering of the syringe system in the pelican case as viewed from the front.

As per the Canadian Reduced Gravity Experiment (CAN-RGX) safety guidelines, all fluid and biological materials are to be securely stored during the flight. A syringe apparatus was built for the purpose of tissue-engineered cartilage storage and media-RNAlater fluid exchange by our engineering team. This apparatus includes 12 groups of 3 syringes connected by tubing and a 3-position valve. The syringes were secured in a custom 3D printed casing that was secured to the bottom of a pelican case (Fig. 2). The syringes containing the engineered cartilage samples each have a heating element wrapped around the body of the syringe and maintained a temperature of 37 °C for the duration of the flight. The temperature output of these heating elements was closely monitored during the experiment. The cartilage samples were terminated with RNAlater by manually manipulating the valve position and pushing the syringe plungers sequentially.

### Parabolic Flight

**Fig. 3:**
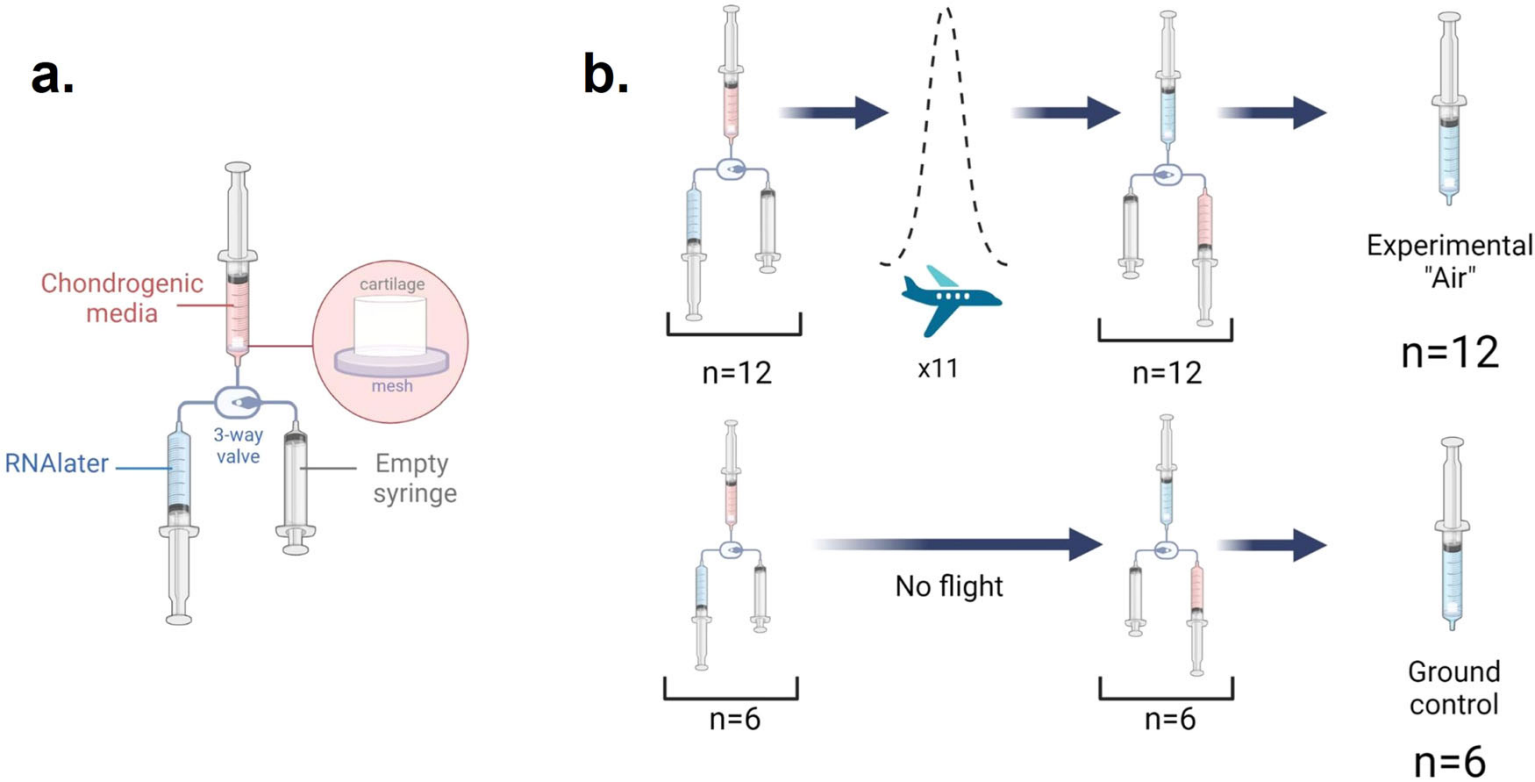
Illustration of one syringe system. **a** Labelled diagram of one syringe system sub-unit containing three syringes connected by tubing and 3-position valve. The top syringe contains bioengineered cartilage sample, a mesh filter, and chondrogenic media. The bottom left syringe contains RNAlater. The bottom right syringe is empty. **b** The top row represents our 12 experimental groups undergoing eleven parabolic flight maneuvers before being terminated by RNAlater. The bottom row represents our six ground control samples remaining stationary on the ground during the period of the parabolic flight maneuvers and being terminated by RNAlater at the same time as the experimental group.

The parabolic flight was conducted in the Falcon 20 shuttle by the National Research Council of Canada. Our samples cycled through 11 parabolas on the National Research Council’s Falcon-20 aircraft (Government of Canada, 2021), exposing them to approximately 140 seconds of true microgravity. After levelling off, the bioengineered cartilage was terminated in RNAlater. The ground control samples were terminated in RNAlater at approximately the same time by our ground crew (Fig. 3). The laboratory control samples were also terminated in RNAlater at approximately the same time as well.

### Total RNA extraction, next-generation sequencing, and RT-qPCR

All tissue-engineered constructs intended for RNA sequencing (RNAseq) and RT-qPCR analysis were preserved in Trizol (Life Technologies, USA) immediately upon harvesting and stored at −80 °C until RNA extraction. RNA was extracted and purified from pestle ground constructs using PuroSPIN Total DNA Purification KIT (Luna Nanotech, Canada) following the manufacturer’s protocol. RNA was reversely transcribed into cDNA, and the genes of interest were amplified by quantitative real-time polymerase chain reaction (RT-qPCR) using specific primers (Supplementary Table 1). The expression level of genes of interest was normalized to chosen housekeeping genes (i.e., *B-actin*, *B2M*, and *YWHAZ*) based on the coefficient of variation (CV) and M-value as measures of reference gene stability^36^, and the data were presented using the 2^-∆∆CT^ method^37^. Next-generation RNA-sequencing was performed on the Illumina NextSeq 500 platform with paired-end 42 bp × 42 bp reads, and FastQ files were obtained for further bioinformatics analysis.

### Bioinformatics

The processing method of bioinformatics data was described in a previous publication^25^. Next-generation sequencing data were analyzed with Partek® Flow® software (Version 10.0.21.0302, Copyright © 2021, Partek Inc., St. Louis, MO, USA). A quality score threshold of 20 was set to trim the raw input reads from the 3’ end. Trimmed data were then aligned to the reference human genome hg38 using the STAR 2.7.3a aligner and followed by the quantification to a transcript model (hg38-RefSeq Transcripts 94 - 2020-05-01) using the Partek E/M algorithm. A noise reduction filter was applied to exclude genes whose maximum read count was below 50. Quantified and filtered reads were then normalized using the Add: 1.0, TMM, and Log 2.0 methods in sequential order. Statistical analysis was performed using analysis of variance (ANOVA) for sex and treatment. Differentially expressed genes (DEGs) for each comparison were determined by p-value and fold change (FC). Enriched Gene Ontology (GO) terms were identified with Partek, and the top 50 enriched GO terms by p-value were condensed with the online REVIGO tool.

### Statistical analysis

All statistical analyses were performed using SPSS 28.0 software (IBM, Canada). CFU-F data were tested for normality using the Shapiro–Wilk test, and Levene’s test was used to assess the homogeneity of error variances. Unpaired Student t-test was used to determine statistical significance between males and females based on confirmation of normality of data distribution. Statistical significance was considered when *p < 0.05 and **p < 0.005.

### Reporting summary

Further information on research design is available in the Nature Research Reporting Summary linked to this article.

## Results

### Stem cell characterization with the CFU assay

**Fig. 4:**
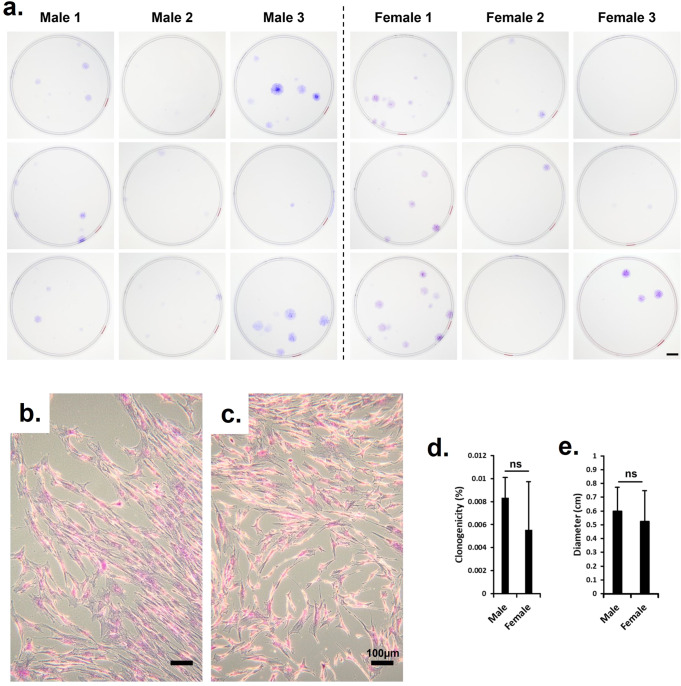
Digital images of CFU-f plates, light microscopy micrographs of plastic adherent stem cell colonies, clonogenicity and colony diameters. **a** Visualization of the CFU-f plates showing the ability of the stem cells to form colonies; **b** Morphology of formed hBMSC colony from male donors; **c** Morphology of formed hBMSC colony from female donors; **d** The Clonogenicity of hBMSC from male and female donors; **e** The mean diameter of hBMSC colonies from male and female donors. All metrics, where relevant, are presented as the mean ± standard deviation. Student t-test unpaired statistics (ns = *p* > 0.05, t-test).

The ability of the mesenchymal stem cells to form adherent cell colonies was assessed with a colony-forming unit fibroblastic (CFU-f) assay. All donors showed colonies in at least 2 out of 3 plates (Fig. 4a). For each donor, 1 × 10^5^ mononucleated cells (MNC) were seeded per plate in triplicates. Figures 4b and c show the morphology of formed hBMSC colonies from male and female donors, respectively. The proportion of seeded MNC resulting in colony formation or clonogenicity was not significantly different between male and female donors (*p* = 0.11; Fig. 4d). Similarly, the mean size of the colonies formed as determined by colony diameters between the male and female donors was not significantly different (*p* = 0.23; Fig. 4e).

### Confirmation of the expression of chondrogenic markers

**Fig. 5:**
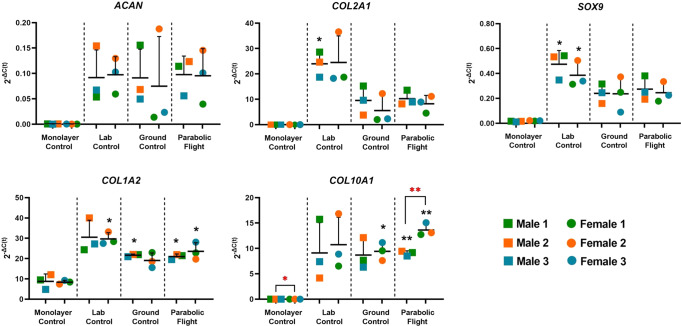
RT-qPCR expression analyses chondrogenic and hypertrophic markers in the laboratory control, ground control, and parabolic flight experimental groups as compared to monolayer cell controls. Statistical significance was considered when *p < 0.05 and **p < 0.005 (t-test) when compared to the same-sex monolayer control group (black) or between sex groups within the same treatment (red). *n* = 3 for both female and male. Each data point represents a single donor.

To further confirm the chondrogenic phenotype of engineered constructs and to evaluate the adoption reactions of constructs to different environmental conditions, RT-qPCR analyses were performed on monolayer cells at the end of the monolayer expansion phase and constructs in all three experimental groups (laboratory control, ground control, and parabolic flight) at the end of parabolic flight. Well-established chondrogenic gene markers (*ACAN, COL1A2, COL2A1*, and *SOX9*) and hypertrophic differentiation marker (*COL10A1*) were measured. As shown in Fig. 5, the average expression level of selected chondrogenic markers increased in all three experimental groups compared to the monolayer control for both male and female donors. Despite the unavoidable donor-to-donor variability, the increased expression of *COL2A1, SOX9, and COL1A2* were significant in certain groups. The expression level of *COL10A1* was significantly higher in the ground control and parabolic flight group for female donors and significantly higher in the parabolic flight group for male donors when compared to the corresponding monolayer group. There was also a significant difference in *COL10A1* expression between male and female monolayer control and parabolic flight groups.

### RNA sequencing dataset overview

**Fig. 6:**
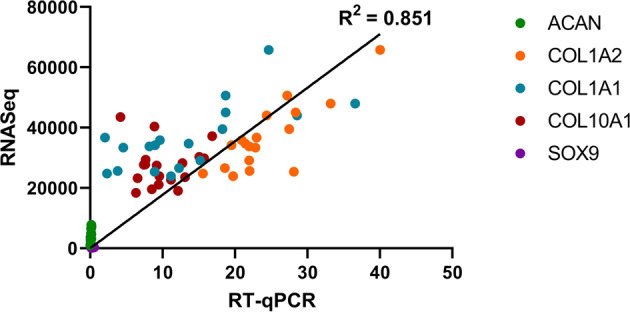
Validation of RNA-Seq data. Linear regression was used to evaluate the correlation between gene expression value measured by RT-qPCR and RNA-Seq analyses.

Bulk transcriptome analysis included the expression profiles of 6 donors (3 females and 3 males), each individually exposed to microgravity via parabolic flight (Air), static ground control (Ground), and static laboratory control (Lab) conditions. After preprocessing as described in the methods, 13,285 genes were preserved for downstream analysis. Figure 6 shows the normalized gene counts from the RNAseq analyses validated against the raw RT-qPCR measurements. An R^2^ value of 0.851 indicates a strong correlation between the two analyses.

### Transcriptome comparison of Ground controls vs Lab controls

We first examined the transcriptome profile differences between the ground control and the laboratory control groups through the RNAseq analysis. After applying the *p* < 0.05, |FC | > 2, total gene counts >250 filters, there were 410 significant differentially expressed genes (DEGs) between the two groups. The top 5 genes with the highest up and downregulated fold changes are shown in Table 2. It is evident, based on the large number of significant DEGs, that the environment experienced by the tissue constructs between the ground control and laboratory control are vastly different. This suggests that laboratory control might not be the ideal control group for the parabolic flight microgravity group, as the different environments, perhaps because of the logistics of the parabolic flight and ground control samples, are confounding that can lead to misguided results. Moving forward, the ground control will be used as the control group for the parabolic flight microgravity.

**Table 2:** Top genes based on fold changes (*p* < 0.05, total counts > 250) in the laboratory control group (Lab) as compared to the ground control group (Ground).

*Gene*	Description	Fold Change	
		Ground/Lab	Air/Ground
*MT1G*	Metallothionein 1G	8.35***	1.60
*TRIB3*	Tribbles Pseudokinase 3	5.81***	−2.32***
*GPNMB*	Glycoprotein Nmb	5.45***	−1.15
*GPR1*	Chemerin Chemokine-Like Receptor 2	4.74***	−1.31
*CRYGS*	Crystallin Gamma S	4.62***	1.08
*LPL*	Lipoprotein Lipase	−6.47***	1.12
*DEPP1*	DEPP1 Autophagy Regulator	−7.14***	−1.35
*HILPDA*	Hypoxia Inducible Lipid Droplet Associated	−7.31***	−1.43
*GPRC5A*	G Protein-Coupled Receptor Class C Group 5 Member A	−8.06***	−1.02
*GALNT15*	Polypeptide N-Acetylgalactosaminyltransferase 15	−11.80***	1.26

***p < 0.001, t-test.

### Transcriptome alteration by microgravity in parabolic flight

Next, we examined the transcriptome profile modulated by microgravity during the parabolic flight on all donors combined. RNA-seq analysis revealed that there was a total of 30 significant differentially expressed genes (DEG) genes (*p* < 0.05 and |FC | > 2) in the parabolic flight group as compared to ground control group (Table 3). The top regulated gene with the highest fold change was *KTI12* (29.7-fold upregulation), and this gene was recently reported to be suppressed in a hypoxia environment (26.8-fold downregulation) in engineered human meniscus^38^. Most other significantly regulated genes were associated with fundamental biological processes, such as cell cycle regulation (*RGCC*, *RHEBL1*), developmental signaling (*GPR18*, *PDGFB*, *FGF21*, and *PCDH1*), or affiliated with the incRNA class (*VASH1-AS1*, *PCNA-AS1*, and *TALAM1*). The OA-related enzyme coding gene *ADAMTS14*, which is required in the formation of type I collagen fibers, was suppressed 2.21-fold by microgravity from parabolic flight.

**Table 3:** All significantly modulated genes (*p* < 0.05, t-test, |FC | > 2) in the microgravity parabolic flight group (Air) as compared to the ground control group (Ground).

*Gene*	Description	P-value: Air vs Ground	Fold Change: Air vs Ground
*KTI12*	KTI12 Chromatin Associated Homolog	2.17E-03	29.65
*HSPA6*	Heat Shock Protein Family A (Hsp70) Member 6	1.39E-02	11.16
*HSPA1B*	Heat Shock Protein Family A (Hsp70) Member 1B	1.24E-04	9.62
*HSPA1A*	Heat Shock Protein Family A (Hsp70) Member 1A	1.77E-04	8.83
*ARC*	Activity Regulated Cytoskeleton Associated Protein	1.70E-03	4.35
*HSPE1-MOB4*	HSPE1-MOB4 Readthrough	1.51E-03	4.28
*GDF10*	Growth Differentiation Factor 10	1.13E-02	2.44
*TALAM1*	TALAM1 Transcript, MALAT1 Antisense RNA	1.43E-02	2.33
*LINC01355*	Long Intergenic Non-Protein Coding RNA 1355	1.45E-05	2.24
*ZBED8*	Zinc Finger BED-Type Containing 8	4.45E-06	2.09
*VASH1-AS1*	VASH1 Antisense RNA 1	5.21E-03	2.06
*ODF3B*	Outer Dense Fiber of Sperm Tails 3B	5.42E-03	2.06
*RGCC*	Regulator Of Cell Cycle	1.01E-06	2.05
*MIR17HG*	MiR-17-92a-1 Cluster Host Gene	1.50E-03	2.04
*PCNA-AS1*	PCNA Antisense RNA 1	6.14E-03	2.02
*SLFN5*	Schlafen Family Member 5	5.62E-08	−2.02
*ANKFN1*	Ankyrin Repeat and Fibronectin Type III Domain Containing 1	1.24E-03	−2.05
*GPR18*	G Protein-Coupled Receptor 18	1.73E-02	−2.11
*KCTD16*	Potassium Channel Tetramerization Domain Containing 16	5.31E-04	−2.13
*RHEBL1*	Ras Homolog Enriched in Brain Like-1 C	3.85E-02	−2.13
*ADAMTS14*	ADAM Metallopeptidase with Thrombospondin Type 1 Motif 14	1.01E-03	−2.21
*FSBP*	Fibrinogen Silencer Binding Protein	4.61E-04	−2.23
*TRIB3*	Tribbles Pseudokinase 3	3.58E-05	−2.32
*PCDH1*	Protocadherin 1	2.29E-02	−2.41
*SPRY1*	Sprouty RTK Signaling Antagonist 1	2.67E-02	−2.41
*RAB4B-EGLN2*	RAB4B-EGLN2 Readthrough	1.26E-02	−2.42
*FGF21*	Fibroblast Growth Factor 21	2.00E-02	−2.48
*LOC102723996*	ICOS Ligand	4.87E-02	−2.60
*RSC1A1*	Regulator Of Solute Carriers 1	4.14E-02	−2.89
*PDGFB*	Platelet Derived Growth Factor Subunit B	1.78E-02	−4.25

**Fig. 7:**
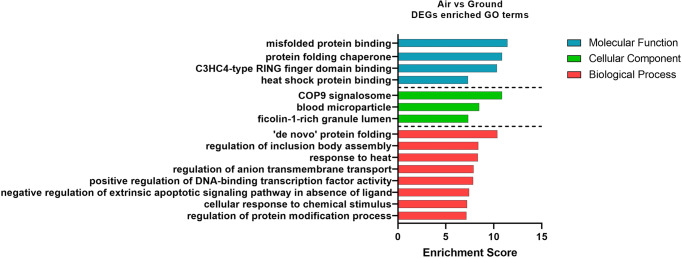
The top non-redundant Gene Ontology (GO) terms enriched by DEG of Air vs Ground. DEGs were identified based on all 6 donors (3 male, 3 female).

The gene ontology database was then used to determine the biological functions of these DEG. The top non-redundant enriched GO terms based on significance level are plotted in Fig. 7. Notably, the most enriched molecular function terms were related to protein activity, as well as the biological process terms “regulation of protein modification process”, and “de novo protein folding.” Other GO terms associated with fundamental biological processes included “cellular response to chemical stimulus”, “positive regulation of DNA-binding transcription factor activity” and “negative regulation of extrinsic apoptotic signaling pathway in absence of ligand.” It is noteworthy that genes from the heat shock protein family, *HSPA1A*, *HSPA1B*, and *HSPA6*, were present in almost every significantly enriched GO term.

### Sex-dependent modulation of transcriptome by Microgravity in parabolic flight

**Fig. 8:**
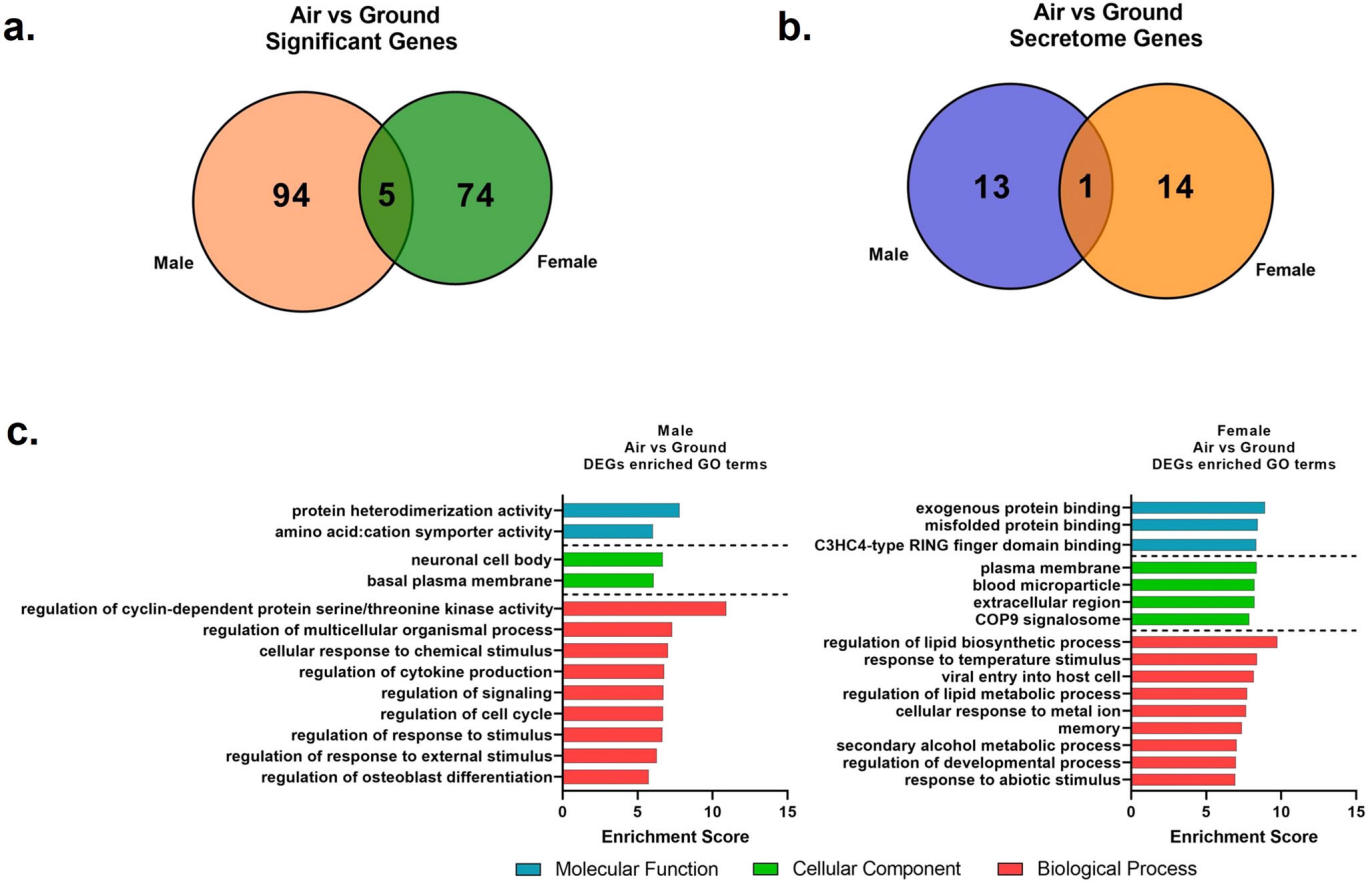
Venn diagram and Gene ontology of differentially expressed genes (DEG) between parabolic flight group vs ground control group for male and female donor cohorts. **a** Venn diagram depicting overlap of differentially expressed genes (DEG) between parabolic flight group vs ground control group for male and female donor cohorts. **b** Venn diagram depicting overlap of differentially expressed secreted gene markers between parabolic flight group vs ground control group for male and female donor cohorts. **c** Top non-redundant Gene Ontology (GO) terms enriched by DEGs of parabolic flight compared to ground controls for male and female donor cohorts.

After assessing the effect of microgravity on all donors combined, we sought to explore the sex-dependent differences in transcriptome profile alteration by separating female and male donors. Table 4 shows the top regulated DEG in parabolic microgravity flight as compared to ground control for male and female donor cohorts. The sex-dependent response was first assessed by overlaying the DEG of females and males together. As shown in Fig. 8a, only a very small portion of DEG (5 genes) was shared between the female and male donor cohorts. The majority of DEG were distinct within each sex group (94 and 74 DEGs for males and females, respectively), indicating a sex-dependent response to parabolic flight microgravity. A similar gene ontology enrichment analysis was conducted within each sex group and the top non-redundant GO terms are presented in Fig. 8.

**Table 4:** A). Top regulated genes based on fold changes in parabolic microgravity (Air) as compared to ground control (Ground) for male and female donor cohorts. B). Top regulated gene markers for secreted factors in Air vs Ground for male and female donor cohorts.

*Gene*	Description	Fold Change: Air/Ground		
		Male	Female	Combined
**A). Top 5 highest up and down regulated genes in Air/Ground for male and female donors**.				
*KTI12*	KTI12 Chromatin Associated Homolog	ns	15.58**	29.65**
*HSPE1-MOB4*	HSPE1-MOB4 Readthrough	ns	13.15***	4.28**
*HSPA1B*	Heat Shock Protein Family A (Hsp70) Member 1B	13.38*	5.61***	9.62***
*ARC*	Activity Regulated Cytoskeleton Associated Protein	2.99*	5.43**	4.35**
*HSPA1A*	Heat Shock Protein Family A (Hsp70) Member 1A	ns	5.20***	8.83***
*IGSF9*	Immunoglobulin Superfamily Member 9	3.06*	−3.29*	ns
*PERM1*	PPARGC1 And ESRR Induced Regulator, Muscle 1	ns	−3.37***	ns
*MT3*	Metallothionein 3	ns	−3.44*	ns
*FGF21*	Fibroblast Growth Factor 21	ns	−3.62*	−2.48*
*LINC02568*	Long Intergenic Non-Protein Coding RNA 2568	ns	−3.66**	ns
*DNAJC25-GNG10*	DNAJC25-GNG10 Readthrough	5.39*	ns	ns
*LOC101929322*	Integrator Complex Subunit 4 Pseudogene	3.39*	ns	1.74*
*GDF10*	Growth Differentiation Factor 10	3.11*	ns	2.44*
*GRID1*	Glutamate Ionotropic Receptor Delta Type Subunit 1	−3.99**	ns	ns
*RHEBL1*	RHEB Like 1	−4.62*	ns	−2.13*
*RN7SK*	RNA Component Of 7SK Nuclear Ribonucleoprotein	−4.97**	ns	ns
*PDGFB*	Platelet Derived Growth Factor Subunit B	−5.14*	ns	−4.25*
*RSC1A1*	Regulator Of Solute Carriers 1	−5.74*	ns	−2.89*
**B). Top regulated gene markers for secreted factors in Air vs Ground for male and female donors**.				
*WNT7B*	Wnt Family Member 7B	ns	2.71*	ns
*CLEC2B*	C-Type Lectin Domain Family 2 Member B	ns	2.45**	ns
*RGMA*	Repulsive Guidance Molecule BMP Co-Receptor A	ns	2.34**	1.58**
*WNT9A*	Wnt Family Member 9A	ns	2.14*	ns
*EFNA1*	Ephrin A1	1.84*	2.06***	1.94***
*ISM2*	Isthmin 2	ns	−2.40*	ns
*PDZD2*	PDZ Domain Containing 2	ns	−2.45*	−1.81*
*STC1*	Stanniocalcin 1	ns	−3.24**	ns
*IGSF9*	Immunoglobulin Superfamily Member 9	3.06*	−3.29*	ns
*FGF21*	Fibroblast Growth Factor 21	ns	−3.62*	−2.48*
*GDF10*	Growth Differentiation Factor 10	3.11*	ns	2.44*
*PKDCC*	Protein Kinase Domain Containing, Cytoplasmic	2.78*	ns	1.37*
*RGCC*	Regulator Of Cell Cycle	2.59**	1.68*	2.05***
*GNRH1*	Gonadotropin Releasing Hormone 1	2.26*	ns	1.75**
*THSD1*	Thrombospondin Type 1 Domain Containing 1	−2.38**	ns	−1.75**
*CABLES1*	Cdk5 And Abl Enzyme Substrate 1	−2.39*	ns	ns
*TSLP*	Thymic Stromal Lymphopoietin	−2.40**	ns	−1.96***
*BRINP1*	BMP/Retinoic Acid Inducible Neural Specific 1	−3.43*	ns	ns
*PDGFB*	Platelet Derived Growth Factor Subunit B	−5.14*	ns	−4.25*

*p < 0.05, **p < 0.01, ***p < 0.001, t-test. ns indicate not significant.

### Identification of encoding genes for secreted factors modulated by microgravity in parabolic flight

Finally, we explored the effect of parabolic flight microgravity on the secretome profiles of all donors combined as well as the sex-stratified groups. Secretome can be defined as the bioactive substrates secreted by the cells as a response to environmental stimulation^39^. A total of 15 secretome genes were significantly regulated for females and 14 for males (Table 4b). Among them, two Wnt-signaling genes *WNT7B* and *WNT9A* were only up-regulated for female donors, by 2.71-fold and 2.14-fold, respectively. One of the immune response-relevant genes, *IGSF9*, was significantly regulated for both males and females in opposite directions (3.06-fold for male and –3.29-fold for female), and two were downregulated only for the males (*THSD1*, -2.38-fold and *TSLP*, -2.40-fold). Two other secretome genes, *RGCC* and *EFNA1*, were commonly upregulated for both female and male.

## Discussion

The motivation to investigate the effects of real microgravity on bioengineered cartilage from human bone marrow MSC (hBMSC) stems from several reasons. One reason is the significant clinical interest in the regenerative potential of hBMSC for cartilage repair^40–51^. Another is the spaceflight microgravity-induced cartilage pathology in mice^52,53^. And yet another is the recently reported sex differences in the effect of simulated microgravity on bioengineered meniscal fibrocartilage^25^. To this end, we embarked on a study investigating the impact of real microgravity via parabolic flight maneuvers on the transcriptome of bioengineered cartilage developed from male and female hBMSC.

First, we established that the mononucleated cells (MNC) of the donor bone marrow aspirates produced characteristic plastic adherent cell colonies that are consistent with fibroblastic features of bone marrow-derived MSC as previously reported by Friedenstein et al.^37^, and as one of the minimal criteria of MSC by the International Society of Cellular Therapy (ISCT). To this end, our data (Figs. 1a and 1b) demonstrated that a proportion of the plated MNC (i.e., clonogenicity) formed cell colonies with fibroblastic morphologies (Fig. 1c) that are consistent with MSC, and the diameter of the colonies matches well with previous measures^33,51^.

We next determined if the hBMSC is inducible towards a chondrocyte phenotype through established in vitro chondrogenic protocol for bioengineered cartilage formation after seeding into porous collagen scaffolds as we have previously reported. The hBMSC, regardless of the biological sex of the donor, differentiated and expressed genes (i.e., *ACAN*, *COL2A1,* and *SOX9*) that are consistent with in vitro chondrogenesis of hBMSC^41,54^. But the monolayer control hBMSC did not express these genes (Fig. 2). However, the chondrogenic gene expression of the resulting bioengineered cartilage was probably influenced by environmental factors even though all samples were maintained in the chondrogenic media until the study’s endpoint. The environmental factors are most likely associated with the less-than-ideal mammalian cell culture conditions during the logistics of the ground control and parabolic flight designated samples relative to the lab control samples, which remained under controlled mammalian cell culture conditions (i.e., 37 °C, 5%CO_2_; 95% humidity). The impact of the environmental factors is particularly evident in the decline of the expression of *COL2A1* and *SOX9* in the ground control and parabolic flight groups relative to the lab control group regardless of the biological sex (Fig. 2). Hence, it is reasonable to compare the data of the parabolic flight with the ground control samples due to their matching environmental conditions.

We validated the gene data from RNASeq with RT-qPCR using a selected panel of genes. Beyond the selected panel of the chondrogenic genes via RT-qPCR (Fig. 2), we next performed a bulk transcriptome profiling of all the samples via RNASeq. The RNASeq and RT-qPCR data correlated positively (R^2^ ~ 0.9; Fig. 3). With the validation, we normalized the parabolic flight (aka “Air”) RNASeq data by the corresponding RNASeq data of the ground control samples. Note that the normalization was performed between samples derived from the same donors. The gene expression changes noted in the articular cartilage of male mice after 30 days of spaceflight were similarly normalized to the gene expression in the articular cartilage of ground control mice^53^.

After the parabolic flight, the top five upregulated genes displayed sex-dependent differences (Table 4a). It is interesting that none of the top five upregulated genes were on the list of genes induced in the articular cartilage of male mice after 30 days of spaceflight microgravity^53^. Most of the upregulated genes were associated with *de novo* protein folding processes based on the GO terms (Fig. 4). The topmost upregulated gene was *KTI12* (Chromatin Associated Homolog), an essential regulatory factor of the Elongator complex, involved in modifying uridine bases in eukaryotic tRNAs^55^ after ten successive parabolas. But this upregulation was notably significant only in bioengineered cartilage from female hBMSC. The molecular basis for this sex-dependent response of *KTI12* to the cumulative parabolas is unclear. Still, given that tRNAs are involved in decoding mRNA transcript into a protein, it is probable that its induction is linked to the decoding of the transcripts of *HSPEI-MOB4* (a conjoined gene^56^ composed of heat shock 10 kDa protein 1 (chaperonin 10)) and MOB4 (MOB family member 4, phocein) and *HSPA1A* (Heat Shock Protein Family A (Hsp70) Member 1A) given their respective second and fifth position in the top five upregulated genes with highly significant fold-increases but only in the engineered cartilage from female hBMSC in response to the ten parabolas of microgravity. Interestingly, both *HSPE-MOB4* (GO:0005524 and GO:0016887) and *HSPA1A* encoded proteins have a high affinity for ATP and ATPase activity^57–59^. Given that both *HSPE-MOB4* and *HSPA1A* are associated with genes encoding heat shock family of proteins (HSP) which are known to be involved in protein folding as chaperones and secreted in response to stress to restore the normal folded state, protect cells and mediate repair of damage caused by various stimuli^60,61^, to this end, our data suggests that *HSPA1A*, a member of the ubiquitous Hsp70 family of HSP known to increase cellular tolerance to various stressors, such as heat, ischemia, and hypoxia is preferentially upregulated (>5-fold; Table 4a) by parabolic flight microgravity in the bioengineered cartilage from female hBMSC^61–64^. In contrast, *HSPA1B*, the third of the top five upregulated genes and another member of the HSP70 family of HSP, was preferentially upregulated in response to the ten parabolas of microgravity in cartilage engineered from male hBMSC as supported by its >13-fold increase in transcript expression versus 5.6-fold increase in cartilage from female hBMSC (Table 4a). The molecular basis for the sex-dependent response of the *HSPA1A* and *HSPA1B* to parabolic microgravity is unclear but given that members of Hsp70 family of HSP are mainly involved in the *de novo* synthesis and transport of proteins to restore normal physiological function of cells, and *KTI12* was only upregulated in the female-derived engineered cartilage samples, it is probable that *KTI12* directly or indirectly modulates *HSPA1A*. Interestingly, this possibility is indirectly supported by the findings that human elongation protein 3 (hELP3), a catalytic subunit of the Elongator complex, participates in the transcription elongation of *HSPA1A* in HeLa cells^65^.

*ARC* (Activity Regulated Cytoskeleton-Associated Protein) was the penultimate upregulated gene of the top five most upregulated genes in response to parabolic flight microgravity. Its upregulation was significant and independent of biological sex (Table 4a). But its upregulation was almost 2-fold higher in bioengineered cartilage from female-derived hBMSC. Given that *ARC* encodes ARC protein which regulates actin dynamics and cytoskeletal rearrangements in various cell types^66–68^, our finding of its upregulation, regardless of biological sex, suggests that the parabolic flights of microgravity altered the cytoskeletal structures of the engineered cartilages albeit to greater extent in engineered cartilage from female-derived hBMSC.

We performed secretome analysis to identify proteins predicted to be secreted by the bioengineered cartilage developed from chondrogenically induced hBMSC^39,69^. Our data revealed that regardless of the biological sex origin of the engineered cartilage, *EFNA1* (Ephrin A1) is upregulated to near the same extent between sexes by the ten parabolas of microgravity (Table 4b). This finding perhaps reflects the importance of spatial localization of EphA-ephrin-A signaling within the earliest stages of chondrogenesis and endochondral ossification during skeletal development^69^. *EFNA1* was the fifth of the five topmost upregulated secretome genes after the ten parabolic flights of the study. However, it is interesting to note that the remaining four of the five topmost upregulated secretome genes were only significant in engineered cartilage derived from female-derived hBMSC. *WNT7B*, a gene encoding a member of the WNT family of proteins which are known to be involved with many developmental, physiological, and disease processes in several cells, was the topmost upregulated secretome-associated gene (Table 4b). Its upregulation after parabolic flights may underly the downward trend in chondrogenic markers (*COL2A1* and *SOX9*; Fig. 2) given that *WNT7B* significantly decreased chondrogenic markers (*SOX9*, *ACAN*, and *COL2A1*) in human-induced pluripotent stem cells (hiPSCs) undergoing chondrogenesis^70^. Another member of the WNT family, *WNT9A*, was the fourth of the top five upregulated secretome genes (Table 4b). *WNT9A* has been implicated in the regulation of Indian hedgehog (Ihh) signaling during chondrogenesis^71^. Its role in the regulation of Ihh may explain the significantly higher expression of the chondrocyte hypertrophy marker, *COL10A1*, in engineered cartilage from female-derived hBMSC since Ihh is a driver of chondrocyte hypertrophy^72,73^.

*CLEC2B* (C-Type Lectin Domain Family 2 Member B) was the second most upregulated secretome transcript after the ten parabolas of microgravity flights (Table 4b). Its exact functional role in chondrogenesis is unclear, but its GO terms: GO:0005515, GO:0030246 and GO:0042802, respectively, suggest functional activities in protein-, carbohydrate- and identical protein binding. Its molecular function regarding carbohydrate binding may be of importance in glycosaminoglycan and proteoglycan metabolism during chondrogenesis but this would have to be verified as well as query its sex-dependent modulation as noted herein^74^.

*RMGA* (Repulsive Guidance Molecule BMP Co-Receptor A) was the third of the top five upregulated secretome transcript after the cumulative parabolic flights campaign of this (Table 4b). Its role is perhaps associated with attempts to maintain chondrocyte homeostasis in response to parabolic microgravity-related stress, given that it has been implicated as a co-receptor in TGFβ/BMP signaling pathways related to cartilage homeostasis^75,76^.

In conclusion, our data provide transcriptomic evidence that bioengineered cartilage from the in vitro chondrogenesis of female- and male-derived hBMSC responds to parabolic flight microgravity in a sex-dependent manner. The sex-dependent response is associated with the transcription of members of the Hsp70 family of heat shock proteins.

## Supplementary information

Supplementary Table 1

Reporting Summary

## Data Availability

The datasets presented in this study can be found in online repositories. The names of the repository/repositories and accession number(s) can be found below: GEO and GSE206008.
