# Supplementary material for: Transcriptomic response of bioengineered human cartilage to parabolic flight microgravity is sex-dependent
Source: NPJ Microgravity. 2023 Jan 19;9:5. doi: 10.1038/s41526-023-00255-6 (PMC9852254; doi:10.1038/s41526-023-00255-6)
Supplement: Supplementary file 1 — Supplementary Table 1 [file 41526_2023_255_MOESM1_ESM.docx]

Supplementary Table 1: Gene-specific primer sequences used for RT-qPCR.

| **Gene** | **Forward** | **Reverse** | **GenBank** | **Hyperlink** |
| --- | --- | --- | --- | --- |
| *ACAN* | AGGGCGAGTGGAATGATGTT | GGTGGCTGTGCCCTTTTTAC | NM_001135.3 | <https://www.ncbi.nlm.nih.gov/nuccore/NM_001135.3> |
| *β-actin* | AAGCCACCCCACTTCTCTCTAA | AATGCTATCACCTCCCCTGTGT | NM_001101.4 | <https://www.ncbi.nlm.nih.gov/nuccore/NM_001101.4> |
| *B2M* | TGCTGTCTCCATGTTTGATGTATCT | TCTCTGCTCCCCACCTCTAAGT | NM_004048.3 | <https://www.ncbi.nlm.nih.gov/nuccore/NM_004048.3> |
| *COL1A2* | GCTACCCAACTTGCCTTCATG | GCAGTGGTAGGTGATGTTCTGAGA | NM_000089.3 | <https://www.ncbi.nlm.nih.gov/nuccore/NM_000089.3> |
| *COL2A1* | CTGCAAAATAAAATCTCGGTGTTCT | GGGCATTTGACTCACACCAGT | NM_001844.5 | <https://www.ncbi.nlm.nih.gov/nuccore/NM_001844.5> |
| *COL10A1* | GAAGTTATAATTTACACTGAGGGTTTCAAA | GAGGCACAGCTTAAAAGTTTTAAACA | NM_000493.3 | <https://www.ncbi.nlm.nih.gov/nuccore/NM_000493.3> |
| *SOX9* | CTTTGGTTTGTGTTCGTGTTTTG | AGAGAAAGAAAAAGGGAAAGGTAAGTTT | NM_000346.3 | <https://www.ncbi.nlm.nih.gov/nuccore/NM_000346.3> |
| *YWHAZ* | TCTGTCTTGTCACCAACCATTCTT | TCATGCGGCCTTTTTCCA | NM_003406.3 | <https://www.ncbi.nlm.nih.gov/nuccore/NM_003406.3> |
