# Supplementary material for: Transcriptomic response of bioengineered human cartilage to parabolic flight microgravity is sex-dependent
Source: NPJ Microgravity. 2023 Jan 19;9:5. doi: 10.1038/s41526-023-00255-6 (PMC9852254; doi:10.1038/s41526-023-00255-6)
Supplement: Supplementary file 2 — Reporting Summary [file 41526_2023_255_MOESM2_ESM.pdf]

nature portfolio

Corresponding author(s): Adetola Adesida

Last updated by author(s): Dec 12, 2022

Reporting Summary

Nature Portfolio wishes to improve the reproducibility of the work that we publish. This form provides structure for consistency and transparency in reporting. For further information on Nature Portfolio policies, see our Editorial Policies and the Editorial Policy Checklist.

Statistics

For all statistical analyses, confirm that the following items are present in the figure legend, table legend, main text, or Methods section.

n/a	Confirmed	
☐	☒	The exact sample size (*n*) for each experimental group/condition, given as a discrete number and unit of measurement
☐	☒	A statement on whether measurements were taken from distinct samples or whether the same sample was measured repeatedly
☐	☒	The statistical test(s) used AND whether they are one- or two-sided<br>*Only common tests should be described solely by name; describe more complex techniques in the Methods section.*
☒	☐	A description of all covariates tested
☒	☐	A description of any assumptions or corrections, such as tests of normality and adjustment for multiple comparisons
☐	☒	A full description of the statistical parameters including central tendency (e.g. means) or other basic estimates (e.g. regression coefficient) AND variation (e.g. standard deviation) or associated estimates of uncertainty (e.g. confidence intervals)
☒	☐	For null hypothesis testing, the test statistic (e.g. *F*, *t*, *r*) with confidence intervals, effect sizes, degrees of freedom and *P* value noted<br>*Give P values as exact values whenever suitable.*
☒	☐	For Bayesian analysis, information on the choice of priors and Markov chain Monte Carlo settings
☒	☐	For hierarchical and complex designs, identification of the appropriate level for tests and full reporting of outcomes
☒	☐	Estimates of effect sizes (e.g. Cohen's *d*, Pearson's *r*), indicating how they were calculated

Our web collection on statistics for biologists contains articles on many of the points above.

Software and code

Policy information about availability of computer code

Data collection	Illumina NextSeq 500 platform with paired-end 42bp × 42bp reads, and FastQ files were obtained.
Data analysis	Partek® Flow® software (Version 10.0.21.0302, Copyright © 2021, Partek Inc., St. Louis, MO, USA). All statistical analyses were performed using SPSS 28.0 software (IBM, Canada).

For manuscripts utilizing custom algorithms or software that are central to the research but not yet described in published literature, software must be made available to editors and reviewers. We strongly encourage code deposition in a community repository (e.g. GitHub). See the Nature Portfolio guidelines for submitting code & software for further information.

Data

Policy information about availability of data

All manuscripts must include a data availability statement. This statement should provide the following information, where applicable:

- Accession codes, unique identifiers, or web links for publicly available datasets
- A description of any restrictions on data availability
- For clinical datasets or third party data, please ensure that the statement adheres to our policy

The datasets presented in this study can be found in online repositories. The names of the repository/repositories and accession number(s) can be found below: GEO and GSE206008 [https://identifiers.org/geo/ GSE206008].

Human research participants

Policy information about studies involving human research participants and Sex and Gender in Research.

Reporting on sex and gender	Biological specimens were collected from both male and female.
Population characteristics	N/A
Recruitment	N/A
Ethics oversight	University of Alberta's Health Research Ethics Board-Biomedical Panel (Study ID: Pro00018778)

Note that full information on the approval of the study protocol must also be provided in the manuscript.

Field-specific reporting

Please select the one below that is the best fit for your research. If you are not sure, read the appropriate sections before making your selection.

☒ Life sciences ☐ Behavioural & social sciences ☐ Ecological, evolutionary & environmental sciences

For a reference copy of the document with all sections, see nature.com/documents/nr-reporting-summary-flat.pdf

Life sciences study design

All studies must disclose on these points even when the disclosure is negative.

Sample size	The cells that were used in the study were isolated from three male and three female human donors that were close in age. We were limited in the number of human specimens. No power calculations for sample size determination were performed. The three donors per biological sex enabled us to perform three independent experiment repeats to ensure reproducibility.
Data exclusions	No data was excluded from the study.
Replication	The three donors of study cells per biological sex enabled us to perform three independent experiment repeats to ensure reproducibility.
Randomization	Samples were not randomized into experimental groups as the study was designed to investigate the effect of microgravity on samples from male and female donors. Therefore, we needed to know before hand what samples are from male and female donors.
Blinding	Blinding was not possible to the investigators but the laboratory service that performed the RNA sequencing was blinded to experimental and control groups.

Reporting for specific materials, systems and methods

We require information from authors about some types of materials, experimental systems and methods used in many studies. Here, indicate whether each material, system or method listed is relevant to your study. If you are not sure if a list item applies to your research, read the appropriate section before selecting a response.

Materials & experimental systems

n/a	Involved in the study	
☒	☐	Antibodies
☒	☐	Eukaryotic cell lines
☒	☐	Palaeontology and archaeology
☒	☐	Animals and other organisms
☒	☐	Clinical data
☒	☐	Dual use research of concern

Methods

n/a	Involved in the study	
☒	☐	ChIP-seq
☒	☐	Flow cytometry
☒	☐	MRI-based neuroimaging
